# Comparative Evaluation of Smear Microscopy, Xpert MTB/RIF, Löwenstein-Jensen Culture, and MGIT 960 for the Diagnosis of Pulmonary and Extrapulmonary Tuberculosis in Tangier, Morocco

**DOI:** 10.7759/cureus.109503

**Published:** 2026-05-23

**Authors:** Reda Amrani Souhli, Hajar Sabri, Salma Lazraq, Kaoutar Bahyat, Bilal Gorfti, Karima Rissoul

**Affiliations:** 1 Microbiology Laboratory, Mohammed VI University Hospital Center, Tangier, MAR; 2 Microbiology Department, Faculty of Medicine and Pharmacy, Abdelmalek Essaâdi University, Tangier, MAR

**Keywords:** diagnostic performance, extrapulmonary tuberculosis (eptb), molecular diagnostics, nothern morocco, tuberculosis

## Abstract

Introduction

Tuberculosis (TB) remains a major public health concern, requiring rapid and accurate diagnostic tools to improve patient management and limit transmission. The aim of this study was to assess the diagnostic performance of smear microscopy, Xpert MTB/RIF, and Löwenstein-Jensen culture compared with liquid culture using the Mycobacterial Growth Indicator Tube (MGIT) 960 liquid culture system as the reference standard. A secondary objective was to evaluate rifampicin resistance and compare its detection by Xpert MTB/RIF with phenotypic methods.

Materials and methods

A retrospective diagnostic performance study was conducted over nine months (July 2025 to March 2026), including both pulmonary and extrapulmonary samples. All specimens were processed under biosafety level 3 laboratory conditions and tested in parallel using smear microscopy, Xpert MTB/RIF, and culture on Löwenstein-Jensen medium and in the MGIT 960 liquid culture system. Diagnostic performance parameters, including sensitivity, specificity, positive predictive value, and negative predictive value, were calculated using the MGIT 960 liquid culture system as the reference standard. The corresponding 95% confidence intervals (95% CIs) were calculated using the exact Clopper-Pearson method.

Results

A total of 386 specimens were included in the analysis, comprising 233 pulmonary samples (60.4%) and 153 extrapulmonary samples (39.6%). Overall, *Mycobacterium tuberculosis* complex was detected in 46 cases (11.9%) by liquid culture using the MGIT 960 system. The positivity rate of Xpert MTB/RIF was 49 cases (12.7%), while smear microscopy detected 31 cases (8.0%) and Löwenstein-Jensen culture identified 36 cases (9.3%). Among culture-negative specimens (340/386, 88.1%), 19/340 cases (5.6%) were positive by Xpert MTB/RIF, of which 3/19 (15.8%) were also smear-positive. Compared with liquid culture using the MGIT 960 system as the reference standard, Xpert MTB/RIF demonstrated higher sensitivity than smear microscopy in both pulmonary specimens (63.3% vs 53.3%) and extrapulmonary specimens (68.8% vs 62.5%), while both tests maintained high specificity. Löwenstein-Jensen culture showed a sensitivity of 76.1% and a specificity of 99.7%. High concordance was observed between Xpert MTB/RIF and phenotypic drug susceptibility testing for rifampicin resistance.

Conclusion

An integrated diagnostic approach combining molecular testing, smear microscopy, and complementary culture methods remains essential for optimal TB diagnosis. Xpert MTB/RIF provides rapid and reliable detection, particularly in smear-negative and extrapulmonary specimens, although culture remains indispensable for confirmation and drug susceptibility testing.

## Introduction

Tuberculosis (TB) remains a critical global health challenge; it continues to place an enormous strain on healthcare systems worldwide. Data from the World Health Organization (WHO) indicate that of the 10.8 million individuals who developed TB in 2023, mortality exceeded 1.25 million cases [1]. The high prevalence of the disease among productive adults complicates clinical management and adversely affects treatment outcomes [2].

Accurate and timely laboratory diagnosis is fundamental to effective TB control. While Ziehl-Neelsen (ZN) smear microscopy remains widely accessible and cost-effective, its utility is limited by low sensitivity, particularly in extrapulmonary and paucibacillary cases [3,4]. Mycobacterial culture continues to be the definitive diagnostic gold standard. Although solid Löwenstein-Jensen (LJ) medium was historically the reference method, the Mycobacterial Growth Indicator Tube (MGIT) liquid culture system is now preferred in contemporary comparative research due to its superior sensitivity and reduced time-to-detection [5,6]. The Xpert MTB/RIF assay (Cepheid®, USA), endorsed by the WHO since 2010 for pulmonary TB and extended in 2013 to extrapulmonary specimens, introduced a major diagnostic shift by delivering results within two hours alongside simultaneous rifampicin resistance detection [7].

Despite the increased use of these tools, a limited number of comparable reports, particularly those including both pulmonary and extrapulmonary samples, are available in the literature, especially in Africa, making the present work particularly relevant. The objective of this study was to evaluate the diagnostic performance of smear microscopy, Xpert MTB/RIF, and LJ culture across different specimen types, using the BACTEC™ MGIT™ 960 system (Becton, Dickinson and Company, USA) as the reference standard. In addition, we assessed the rate of rifampicin resistance detected by Xpert MTB/RIF and compared it with phenotypic drug susceptibility testing results.

## Materials and methods

Study design

A retrospective diagnostic performance study was conducted at the microbiology laboratory of the General University Hospital of Tangier, Morocco. Clinical specimens received for the investigation of suspected TB over a nine-month period (July 2025 to March 2026) were included in the analysis.

Study population and specimens 

A total of 386 pulmonary and extrapulmonary specimens were received in the microbiology laboratory. Only specimens from patients who underwent all four diagnostic tests - smear microscopy for acid-fast bacilli, the Xpert MTB/RIF molecular assay, and mycobacterial culture performed on both liquid medium using the MGIT 960 system and solid Löwenstein-Jensen (LJ) medium - were included in the study. Only one specimen per patient was retained for analysis; when multiple specimens were available from the same patient, only the first sample was included. Specimens with incomplete laboratory results for any of these diagnostic methods or contaminated cultures were excluded from the analysis.

Sample processing

All specimens were processed under Biosafety Level 3 conditions. Procedures varied based on the specimen type: Sterile body fluids were processed without decontamination, whereas non-sterile samples underwent NALC-NaOH decontamination. Pulmonary and contaminated extrapulmonary specimens were decontaminated and concentrated before smear microscopy, MGIT, and LJ culture. Cerebrospinal fluid was processed directly without centrifugation or decontamination, while other sterile extrapulmonary fluids were concentrated by centrifugation alone. Urine specimens were first concentrated and subsequently decontaminated. Solid tissue specimens, including lymph node biopsies and other surgically resected tissues, were first minced with a sterile scalpel and then ground in a sterile mortar with a small volume of sterile saline until a homogeneous suspension was obtained. Given that these specimens are collected aseptically, one portion of the homogenate was inoculated directly onto culture media without decontamination. A second portion was decontaminated with NALC-NaOH when surface contamination could not be excluded. 

Smear microscopy

Direct smear microscopy for the detection of acid-fast bacilli (AFB) was performed using the ZN staining method on processed specimens. Microscopic examination was performed under oil immersion (×1000 magnification). Results were interpreted using a semi-quantitative grading system according to the number of bacilli observed per microscopic field.

Xpert MTB/RIF assay

Molecular detection of MTBC DNA and rifampicin resistance was performed using the Xpert MTB/RIF assay (Cepheid, Sunnyvale, CA, USA) according to the manufacturer's instructions. The processed sample was mixed with the provided sample reagent at a 1:2 ratio, vortexed briefly, and incubated at room temperature for 10 minutes before transfer to the cartridge.

Results were reported as MTBC detected or not detected, with rifampicin resistance status when applicable; the automated run was initiated immediately after loading.

Mycobacterial culture

Culture on solid LJ medium was performed and incubated according to standard laboratory procedures. Positive cultures were identified based on colony morphology and confirmed by ZN smear microscopy.

Culture in liquid medium was performed using the BACTEC™ MGIT™ 960 system, with incubation and automated monitoring following standard procedures as previously described. Positive MGIT cultures were confirmed by ZN smear microscopy, and MTBC was identified using a rapid MPT64 antigen immunochromatographic test (BD MGIT™ TBc Identification Test, Becton, Dickinson and Company, Sparks, MD, USA) [8]. Only MPT64-positive isolates were included in the study. Phenotypic drug susceptibility testing (DST) was performed on all MTBC isolates recovered in culture according to routine laboratory procedures.

Quality control

Quality control measures were implemented at each stage of the laboratory workflow in accordance with standard operating procedures and international guidelines. For AFB smear microscopy, ZN staining was performed according to standardized protocols, with positive and negative control smears included in each staining batch to verify staining quality and decolorization performance. Selected smears were independently reviewed by a second experienced technician as part of internal quality assurance. For the Xpert MTB/RIF assay, all tests were performed according to the manufacturer's recommendations, with quality control ensured through the integrated Sample Processing Control (SPC) and Probe Check Control (PCC) embedded in each cartridge; instrument calibration and maintenance were performed according to the manufacturer's schedule. For Löwenstein-Jensen solid culture, each new batch was inspected for sterility, appropriate coagulation, and color uniformity before routine use, and positive and negative controls were included to verify growth performance. For MGIT liquid culture, each new lot of medium and enrichment supplement underwent validation prior to use, and positive and negative controls were processed to assess system performance. For both culture methods, controls were processed concurrently with clinical specimens, and contamination rates, incubation conditions, and time-to-detection values were routinely recorded as part of ongoing internal quality assurance.

Statistical analysis

Data were extracted from the laboratory database and analyzed using the Statistical Package for the Social Sciences (SPSS), version 26.0 (IBM Corp., Armonk, NY, USA). Categorical variables were expressed as numbers and percentages. The positivity rate of each diagnostic method was calculated based on the number of specimens tested. Diagnostic performance parameters, including sensitivity, specificity, positive predictive value (PPV), and negative predictive value (NPV), were calculated using MGIT liquid culture as the reference standard. The corresponding 95% confidence intervals (95% CIs) were calculated using the exact Clopper-Pearson method.

Ethical considerations

This study was based exclusively on pre-existing anonymized laboratory data, with no additional sample collection, no patient contact, and no modification of patient management. It was conducted in accordance with the Declaration of Helsinki and received a posteriori approval from the Hospital-University Ethics Committee of Tangier (Comité d’Éthique Hospitalo-universitaire de Tanger; ref. no. 257/26) on May 12, 2026. In accordance with national regulations and the non-interventional nature of the study, the requirement for informed consent was waived by the ethics committee.

## Results

A total of 386 specimens were analyzed. The mean age of the patients was 46.6 ± 21.2 years, with a range from three months to 91 years. Among them, 222 were men (57.5%) and 164 were women (42.5%), resulting in a male-to-female ratio of 1.35. Thirty-six patients (9.3%) were aged <15 years, 228 (59.1%) were between 15 and 60 years, and 122 (31.6%) were aged >60 years. Most specimens were pulmonary (233; 60.4%), while extrapulmonary specimens accounted for 153 (39.6%). Table 1 summarizes the demographic characteristics of the study population.

**Table 1:** Demographic characteristics of the patients included in the study

Characteristics	Patients (N = 386)	Percentage %
Gender		
Male	222	57.5
Female	164	42.5
Age group		
<15 years	36	9.3
15-60 years	228	59.1
>60 years	122	31.6
Departments		
Pneumology	190	49.2
Other medical services	116	30.1
Surgical services	46	11.9
Pediatrics	27	7.0
ICU	7	1.8
Specimen type		
Pulmonary	233	60.4
Extrapulmonary	153	39.6

Of the 386 specimens tested, the Xpert MTB/RIF assay showed the highest overall positivity rate of 12.7% (49/386) compared with the other diagnostic methods. In pulmonary specimens, the positivity rate was 10.3% (24/233), while a higher detection rate was observed in extrapulmonary specimens at 16.3% (25/153). The assay demonstrated notably increased detection in tissue biopsies (50.0%), lymph node specimens (27.3%), and pus samples (25.0%).

By contrast, smear microscopy showed the lowest overall positivity rate of 8.0% (31/386), with a notably higher positivity in cerebrospinal fluid (17.6%) compared with the Xpert MTB/RIF assay (5.9%).

Culture methods showed intermediate diagnostic yields across all specimens, with a positivity rate of 9.3% (36/386) for LJ culture and 11.9% (46/386) for MGIT liquid culture (Table 2). 

**Table 2:** Positivity rates of smear microscopy, Xpert MTB/RIF, Löwenstein-Jensen culture, and MGIT liquid culture according to the specimen type. MGIT: Mycobacterial Growth Indicator Tube

Category and specimen type	Total	Smear microscopy positive n (%)	Smear microscopy negative n (%)	Xpert MTB/RIF positive n (%)	GeneXpert MTB/RIF negative n (%)	LJ culture positive n (%)	LJ culture negative n (%)		MGIT culture positive n (%)	MGIT culture negative n (%)
Sputum	223	17 (7.6)	206 (92.4)	21 (9.4)	202 (90.6)	21 (9.4)	202 (90.6)	27 (12.1)		196 (87.9)
Bronchial aspirate	9	0	9 (100.0)	2 (22.2)	7 (77.8)	2 (22.2)	7 (77.8)	2 (22.2)		7 (77.8)
Bronchoalveolar lavage (BAL)	1	1 (100.0)	0	1 (100.0)	0	0	1 (100.0)	1 (100.0)		0
Pulmonary specimens	233	18 (7.7)	215 (92.3)	24 (10.3)	209 (89.7)	23 (9.9)	210 (90.1)	30 (12.9)		203 (87.1)
Puncture fluid	57	3 (5.4)	54 (94.6)	8 (14.0)	49 (86.0)	6 (10.5)	51 (89.5)	6 (10.5)		51 (89.5)
Cerebrospinal fluid (CSF)	17	3 (17.6)	14 (82.4)	1 (5.9)	16 (94.1)	0 (0.0)	17 (100.0)	1 (5.9)		16 (94.1)
Pus	12	2 (16.7)	10 (83.3)	3 (25.0)	9 (75.0)	2 (16.7)	10 (83.3)	3 (25.0)		9 (75.0)
Lymph node	11	2 (18.2)	9 (81.8)	3 (27.3)	8 (72.7)	2 (18.2)	9 (81.8)	3 (27.3)		8 (72.7)
Tissue biopsy	16	1 (6.3)	15 (93.8)	8 (50.0)	8 (50.0)	1 (6.3)	15 (93.8)	1 (6.3)		15 (93.8)
Urine	3	0 (0.0)	3 (100.0)	0 (0.0)	3 (100.0)	0 (0.0)	3 (100.0)	0 (0.0)		3 (100.0)
Other	37	2 (5.4)	35 (94.6)	2 (5.4)	35 (94.6)	2 (5.4)	35 (94.6)	2 (5.4)		35 (94.6)
Extrapulmonary specimens	153	13 (8.5)	140 (91.5)	25 (16.3)	128 (83.7)	13 (8.5)	140 (91.5)	16 (10.5)		137 (89.5)
Total	386	31 (8.0)	355 (92.0)	49 (12.7)	337 (87.3)	36 (9.3)	350 (90.7)	46 (11.9)		340 (88.1)

Among the MGIT-negative specimens, 19 were positive by GeneXpert MTB/RIF (10.2%), of which three (15.8%) were also smear-positive, including two sputum samples and one tissue biopsy. LJ culture yielded only one positive case among the extrapulmonary specimens (1/137, 0.7%). Of note, two smear-positive specimens from CSF had negative results for Xpert MTB/RIF and MGIT. The results of smear microscopy, GeneXpert MTB/RIF, and LJ culture in MGIT-negative specimens are summarized in Table 3.

**Table 3:** Distribution of positive results by smear microscopy, Xpert MTB/RIF, and LJ culture in MGIT-negative specimens by specimen type BAL: bronchoalveolar lavage, CSF: cerebrospinal fluid, LJ: Löwenstein-Jensen, MGIT: Mycobacterial Growth Indicator Tube, NA: not applicable

Specimen type	MGIT-negative samples n	Smear positive n (%)	GeneXpert positive n (%)	LJ positive n (%)
Sputum	196	2 (1.0%)	5 (2.6%)	0 (0.0%)
Bronchial aspirate	7	0 (0.0%)	0 (0.0%)	0 (0.0%)
BAL	0	NA	NA	NA
Pulmonary specimens	203	2 (1.0%)	5 (2.5%)	0 (0.0%)
Puncture fluid	51	0 (0.0%)	4 (7.8%)	1 (2.0%)
CSF	16	2 (12.5%)	1 (6.3%)	0 (0.0%)
Pus	9	0 (0.0%)	1 (11.1%)	0 (0.0%)
Lymph node	8	0 (0.0%)	1 (12.5%)	0 (0.0%)
Tissue biopsy	15	1 (6.7%)	7 (46.7%)	0 (0.0%)
Urine	3	0 (0.0%)	0 (0.0%)	0 (0.0%)
Other	35	0 (0.0%)	0 (0.0%)	0 (0.0%)
Extrapulmonary specimens	137	3 (2.2%)	14 (10.2%)	1 (0.7%)
Total	340	5 (1.5%)	19 (5.6%)	1 (0.3%)

The detailed sensitivity, specificity, and positive and negative predictive values of smear microscopy, GeneXpert MTB/RIF, and LJ culture, using MGIT culture as the reference standard, are presented for each specimen category in Tables 4-5.

**Table 4:** Diagnostic performance of smear microscopy and GeneXpert MTB/RIF compared with MGIT culture according to specimen type MGIT: Mycobacterial Growth Indicator Tube, CI: confidence interval; SE: sensitivity; SP: specificity; PPV: positive predictive value; NPV: negative predictive value; NA: not applicable due to absence of positive or negative MGIT cases

Type of samples	Smear microscopy SE % (95% CI)	Smear microscopy SP % (95% CI)	Smear microscopy PPV % (95% CI)	Smear microscopy NPV % (95% CI)	Xpert MTB/RIF SE % (95% CI)	Xpert MTB/RIF SP % (95% CI)	Xpert MTB/RIF PPV % (95% CI)	Xpert MTB/RIF NPV % (95% CI)
Sputum	55.6% (35.3–74.5%)	99.0% (96.4–99.9%)	88.2% (63.6–98.5%)	94.2% (89.9–97.1%)	59.3% (38.8–77.6%)	97.4% (94.2–99.1%)	76.2% (52.8–91.8%)	94.6% (90.3–97.3%)
Bronchial aspirate	NA	100% (59.0–100.0%)	NA	77.8% (40.0–97.2%)	100% (15.8–100.0%)	100% (59.0–100.0%)	100% (15.8–100.0%)	100% (59.0–100.0%)
Bronchoalveolar lavage	100% (2.5–100.0%)	NA	100% (2.5–100.0%)	NA	100% (2.5–100.0%)	NA	100% (2.5–100.0%)	NA
Pulmonary samples	53.3% (34.3–71.7%)	99.0% (96.5–99.9%)	88.9% (65.3–98.6%)	93.5% (89.4–96.4%)	63.3% (43.9–80.1%)	97.5% (94.3–99.2%)	79.2% (57.8–92.9%)	94.7% (90.8–97.3%)
Cerebrospinal fluid	100% (2.5–100.0%)	87.5% (61.7–98.4%)	33.3% (0.8–90.6%)	100% (76.8–100.0%)	0.0% (0.0–97.5%)	93.8% (69.8–99.8%)	0.0% (0.0–97.5%)	93.8% (69.8–99.8%)
Lymph node specimens	66.7% (9.4–99.2%)	100% (63.1–100.0%)	100% (15.8–100.0%)	88.9% (51.8–99.7%)	66.7% (9.4–99.2%)	87.5% (47.3–99.7%)	66.7% (9.4–99.2%)	87.5% (47.3–99.7%)
Tissue biopsies	0.0% (0.0–97.5%)	93.3% (68.1–99.8%)	0.0% (0.0–97.5%)	93.3% (68.1–99.8%)	100% (2.5–100.0%)	53.3% (26.6–78.7%)	12.5% (0.3–52.7%)	100% (63.1–100.0%)
Pus	66.7% (9.4–99.2%)	100% (66.4–100.0%)	100% (15.8–100.0%)	90.0% (55.5–99.7%)	66.7% (9.4–99.2%)	88.9% (51.8–99.7%)	66.7% (9.4–99.2%)	88.9% (51.8–99.7%)
Puncture fluid	50.0% (11.8–88.2%)	100% (93.0–100.0%)	100% (29.2–100.0%)	94.4% (84.6–98.8%)	66.7% (22.3–95.7%)	92.2% (81.1–97.8%)	50.0% (15.7–84.3%)	95.9% (86.0–99.5%)
Urine	NA	100% (29.2–100.0%)	NA	100% (29.2–100.0%)	NA	100% (29.2–100.0%)	NA	100% (29.2–100.0%)
Others	100% (15.8–100.0%)	100% (90.0–100.0%)	100% (15.8–100.0%)	100% (90.0–100.0%)	100% (15.8–100.0%)	100% (90.0–100.0%)	100% (15.8–100.0%)	100% (90.0–100.0%)
Extrapulmonary samples	62.5% (35.4–84.8%)	97.8% (93.7–99.5%)	76.9% (46.2–95.0%)	95.7% (90.9–98.4%)	68.8% (41.3–89.0%)	89.8% (83.5–94.3%)	44.0% (24.4–65.1%)	96.1% (91.1–98.7%)
Total	56.5% (41.1–71.1%)	98.5% (96.6–99.5%)	83.9% (66.3–94.5%)	94.4% (91.4–96.6%)	65.2% (49.8–78.6%)	94.4% (91.4–96.6%)	61.2% (46.2–74.8%)	95.3% (92.5–97.3%)

**Table 5:** Diagnostic performance of Löwenstein–Jensen culture compared with MGIT culture according to specimen type CI: confidence interval; LJ: Löwenstein–Jensen; NA: not applicable due to the absence of positive or negative MGIT cases

Type of samples	LJ SE % (95% CI)	LJ SP % (95% CI)	LJ PPV % (95% CI)	LJ NPV % (95% CI)
Sputum	77.8% (57.7–91.4%)	100% (98.1–100.0%)	100% (83.9–100.0%)	97.0% (93.7–98.9%)
Bronchial aspirate	100% (15.8–100.0%)	100% (59.0–100.0%)	100% (15.8–100.0%)	100% (59.0–100.0%)
Bronchoalveolar lavage	0.0% (0.0–97.5%	NA	NA	0.0% (0.0–97.5%
Pulmonary samples	76.7% (57.7–90.1%)	100% (98.2–100.0%)	100% (85.2–100.0%)	96.7% (93.3–98.7%)
Cerebrospinal fluid	0.0% (0.0–97.5%	100% (79.4–100.0%)	NA	94.1% (71.3–99.9%
Lymph node specimens	66.7% (9.4–99.2%)	100% (63.1–100.0%)	100% (15.8–100.0%)	88.9% (51.8–99.7%)
Tissue biopsies	100% (2.5–100.0%)	100% (78.2–100.0%)	100% (2.5–100.0%)	100% (78.2–100.0%)
Pus	66.7% (9.4–99.2%)	100% (66.4–100.0%)	100% (15.8–100.0%)	90.0% (55.5–99.7%)
Puncture fluid	83.3% (35.9–99.6%)	98.0% (89.6–100.0%)	83.3% (35.9–99.6%)	98.0% (89.6–100.0%)
Urine	NA	100% (29.2–100.0%)	NA	100% (29.2–100.0%)
Others	100% (15.8–100.0%)	100% (90.0–100.0%)	100% (15.8–100.0%)	100% (90.0–100.0%)
Extrapulmonary samples	75.0% (47.6–92.7%)	99.3% (96.0–100.0%)	92.3% (64.0–99.8%)	97.1% (92.7–99.2%)
Total	76.1% (61.2–87.4%)	99.7% (98.4–100.0%)	97.2% (85.5–99.9%)	96.9% (94.5–98.5%)

Diagnostic results for rifampicin resistance by GeneXpert MTB/RIF among the 49 positive samples showed a detection rate of 2.0% (n = 1). Of the 46 MGIT culture-positive isolates, two (4.3%) were rifampicin-resistant; one was concordant with the GeneXpert MTB/RIF result, while the second yielded an indeterminate result by GeneXpert MTB/RIF.

Regarding the other antibiotics tested, isoniazid resistance was observed in six isolates (13.0%), while resistance to ethambutol and streptomycin was detected in two (4.3%) and one isolate (2.2%), respectively (Table 6).

**Table 6:** Drug resistance profiles obtained by MGIT and GeneXpert MTB/RIF NT: not tested

Drug	MGIT (n = 46) n (%)	GeneXpert MTB/RIF (n = 49) n (%)
Rifampicin	2 (4.3%)	1 (2.0%)
Isoniazid	6 (13.0%)	NT
Ethambutol	2 (4.3%)	NT
Streptomycin	1 (2.2%)	NT

## Discussion

In our study, the majority of patients were between 15 and 60 years, with a predominance of males. This distribution is consistent with epidemiological data showing that TB disproportionately affects economically active populations, with important implications for disease transmission and healthcare burden [1,2]. The high proportion of extrapulmonary specimens (39.6%) reflects a broader epidemiological transition documented in Morocco, where the proportion of extrapulmonary TB among all notified cases rose from approximately 23% to nearly 47% between 1980 and 2022 [9]. This shift reinforces the clinical imperative for diagnostic tools capable of detecting TB across a wide spectrum of specimen types, beyond the traditional sputum-centric paradigm.

The detection rates were variable, ranging from 8.0% for smear microscopy to 12.7% for Xpert MTB/RIF. These results are consistent with the literature, which shows that molecular diagnostics offer higher diagnostic yields than microscopy, particularly in smear-negative and paucibacillary specimens [10-12]. In Morocco, Mechal et al. reported a higher overall Xpert MTB/RIF positivity rate of 20.59% and a smear positivity rate of 12.88% from a Rabat military teaching hospital cohort [13], likely reflecting differences in patient selection, disease severity, and the proportion of immunocompromised individuals between the two populations.

The higher positivity rate of Xpert MTB/RIF in extrapulmonary samples (16.3%) compared with pulmonary samples (10.3%) is particularly noteworthy. In tissue biopsies, the detection rate reached 50.0%, consistent with Zhao J et al., who demonstrated that GeneXpert achieved the highest recovery rate in biopsy samples (82.7%) compared with liquid culture (73.3%) when both methods were applied to the same samples [14]. The superior performance of nucleic acid amplification in tissue may be attributed to the capacity of PCR-based methods to detect non-viable bacilli and fragmented DNA present in granulomatous lesions where viable bacillary load is insufficient for culture recovery [15]. This is particularly relevant in caseous necrotic tissue, where culture yield is often disproportionately low relative to the degree of histological inflammation.

Similarly, the detection rate of 27.3% in lymph node specimens is clinically meaningful, though it falls below pooled meta-analytic estimates of 83-85% sensitivity for lymph node TB when using fine-needle aspirates [16]. The discrepancy is most likely explained by specimen quality, processing variability, and the small number of lymph node samples (n = 11) in our study, which limits statistical reliability. Sensitivity for lymph node TB is known to be strongly influenced by specimen freshness and the adequacy of sample homogenization prior to cartridge loading [12]. 

Smear microscopy showed a small proportion of positive results in culture-negative specimens (1.5% overall), consistent with previously reported rates of 1-5% in routine clinical practice [17]. Such smear-positive, culture-negative results are commonly attributed to the presence of non-viable or damaged bacilli that remain detectable microscopically but are unable to replicate in culture. Prior antibiotic or anti-tubercular treatment may further reduce bacterial viability, leading to false-negative culture results. Additionally, smear microscopy does not differentiate between *Mycobacterium tuberculosis *complex and non-tuberculous mycobacteria (NTM), which may also contribute to these discordant results [17]. Among the 19 Xpert MTB/RIF-positive, MGIT-negative cases in our study, only 3 (15.8%) were also smear-positive, further supporting the presence of a low bacillary burden in these specimens. Comparable results were reported by Mechal et al. with a culture-negative Xpert-positive rate of 10.61% [13] and by Iram et al., who described a similar 15% rate in an endemic setting [18]. Discordance in this direction may be explained by specimen quality, bacillary load heterogeneity, sampling technique, or prior anti-tubercular therapy.

Furthermore, two smear-positive CSF specimens were negative by both Xpert MTB/RIF and MGIT culture. This discordance warrants careful interpretation rather than dismissal as an analytical artefact. Several mechanisms may explain these results. Smear microscopy in CSF carries a well-recognized risk of false-positive results due to morphological artefacts from cellular debris, lipid droplets, and non-tuberculous acid-fast organisms that may mimic AFB on ZN staining [19]. A systematic review and meta-analysis of CSF AFB smear for tuberculous meningitis diagnosis documented a pooled sensitivity of only 8% (95% CI 3-21%) against a microbiological reference standard, with substantial heterogeneity in both sensitivity and specificity across studies, and false-positive rates that are more likely when highly concentrated CSF pellets are read under suboptimal microscopy conditions [20]. 

The diagnostic advantage of Xpert MTB/RIF over smear microscopy was quantifiable: overall sensitivity increased by approximately 8.7 percentage points (65.2% vs. 56.5%), with a more pronounced advantage in extrapulmonary specimens (68.8% vs. 62.5%), underscoring the clinical utility of molecular detection in paucibacillary conditions. These findings align with published literature: a meta-analysis by Li et al. (2017) reported Xpert MTB/RIF sensitivities of 97-99% in smear-positive specimens and 68-73% in smear-negative cases, while Opota et al. (2019) observed sensitivities of 100% in smear-positive and 66.7% in smear-negative specimens [21,22]. The PPV for Xpert MTB/RIF is strongly influenced by the local prevalence of TB [7]. Conversely, the high NPV observed in our study (94.7% for pulmonary specimens; 96.1% for extrapulmonary specimens) confirms the practical utility of Xpert MTB/RIF as a rule-out test, consistent with WHO data where the NPV exceeds 99% regardless of TB prevalence [7]. These results support WHO TB diagnostic guidelines and confirm the relevance of Xpert MTB/RIF as a primary diagnostic tool [1], while reminding clinicians that a positive result should be interpreted within its specific epidemiological context.

The higher positivity rate of MGIT liquid culture (11.9%) compared with LJ solid culture (9.3%), and the LJ culture sensitivity of 76.1% and specificity of 99.7% relative to MGIT as the reference standard, are consistent with the established superiority of liquid culture systems. Large comparative studies have demonstrated that MGIT detects significantly more positive samples than LJ culture (48.8% versus 41.3%, respectively, in one series), with a substantially shorter mean time to detection [23]. In a dedicated pulmonary TB series, MGIT achieved a recovery rate of 92.3% compared with 69.2% for LJ, with a mean time to positivity of approximately 12 versus 30 days, respectively [24]. The high specificity and PPV of LJ culture (97.2%) underline its continued diagnostic value as a complementary culture method. Isolate recovery may vary between liquid and solid media due to differences in inhibitor tolerance, contamination rates, and the growth dynamics of different mycobacterial strains. Neither method alone detected all positive cases in our study, supporting the parallel use of both culture systems to maximize diagnostic yield, as has been recommended by several authors [24,25]. 

The rifampicin resistance rate of 4.3% by MGIT culture and 2.0% by Xpert MTB/RIF in our were higher than the WHO estimates for Morocco (approximately 1% of new TB cases) [1], and the small absolute number of resistant cases limits the precision of our estimate. The indeterminate Xpert result for rifampicin illustrates a mechanistically well-characterised limitation of probe-based resistance detection. Indeterminate results occur when probe hybridisation is disrupted by mutations outside the canonical rifampicin resistance-determining region (RRDR) hotspots targeted by the assay's five probes [26]. These "disputed" mutations, including those at codons 432 and 446, may represent low-level resistance or heteroresistance, and their clinical significance relative to phenotypic breakpoints remains incompletely defined [26]. Indeterminate Xpert results should therefore be reflexed to phenotypic DST.

The isoniazid resistance rate of 13.0% is consistent with previously reported Moroccan data: Mechal et al. reported 10.34% in Rabat and El Baghdadi et al. documented 11.4% in Casablanca [13,27]. Isoniazid mono-resistance, even in the absence of rifampicin co-resistance, is associated with higher rates of treatment failure, increased risk of progression to multidrug-resistant TB if standard first-line regimens are used unadjusted, and constitutes an under-recognized diagnostic gap in settings relying solely on Xpert MTB/RIF's rifampicin-centric resistance screening [28].

Resistance to ethambutol (4.3%) and streptomycin (2.2%) in our study is consistent with global WHO data, indicating that resistance to these agents remains less frequent than to isoniazid and Rifampicin [1]. The relatively low ethambutol resistance rate likely reflects the protective effect of combination regimens, which may limit selective pressure on ethambutol-specific gene targets. The low streptomycin resistance rate is consistent with its declining use in current treatment protocols, as streptomycin resistance is more frequently associated with prior treatment exposure than with recent transmission [1,29,30].

Limitations

First, the retrospective single-centre design may limit the generalisability of the findings. Second, only samples submitted for routine diagnostic testing were included, which may have introduced selection bias. Third, the high proportion of extrapulmonary and paucibacillary samples, together with the small number of certain sample types, may have influenced diagnostic performance and limited subgroup analyses. In addition, information on prior antibiotic or anti-tubercular treatment was unavailable, which may have contributed to false-negative culture results and discordance with Xpert MTB/RIF. Finally, smear microscopy cannot distinguish *Mycobacterium tuberculosis* complex from non-tuberculous mycobacteria, which may also explain some discordant findings.

## Conclusions

This study reinforces the value of an integrated, multi-method diagnostic strategy for tuberculosis across a heterogeneous clinical population. Among the methods evaluated, Xpert MTB/RIF demonstrated the highest overall diagnostic yield and emerged as the most effective frontline tool, particularly in extrapulmonary specimens where culture-based methods are frequently limited. While sensitivity remained constrained in paucibacillary samples, Xpert MTB/RIF proved reliable across diverse specimen types and clinical presentations. MGIT liquid culture outperformed LJ solid culture, yet both methods contributed complementary diagnostic value, supporting their concurrent use in routine practice. Taken together, these findings confirm that no single method is sufficient in isolation, and that combining molecular testing, liquid culture, and conventional techniques is essential to optimize case detection and resistance assessment in high-burden settings.
